# Dopamine Transporter Deficiency Syndrome (DTDS): Expanding the Clinical Phenotype and Precision Medicine Approaches

**DOI:** 10.3390/cells12131737

**Published:** 2023-06-28

**Authors:** Joanne Ng, Serena Barral, Simon N. Waddington, Manju A. Kurian

**Affiliations:** 1 Gene Transfer Technology Group, EGA UCL Institute for Women’s Health, University College London, London WC1E 6HX, UK; j.ng@ucl.ac.uk (J.N.); s.waddington@ucl.ac.uk (S.N.W.); 2 Genetic Therapy Accelerator Centre, Queens Square Institute of Neurology, University College London, London WC1N 3BG, UK; 3 Developmental Neurosciences, Zayed Centre for Research into Rare Disease in Children, GOS UCL Institute of Child Health, University College London, London WC1N 1DZ, UK; s.barral@ucl.ac.uk; 4 Wits/SAMRC Antiviral Gene Therapy Research Unit, Faculty of Health Sciences, University of the Witwatersrand, Johannesburg 2193, South Africa; 5 Paediatric Neurology, Great Ormond Street Hospital for Children, London WC1N 3JH, UK

**Keywords:** dopamine transporter deficiency syndrome, infantile parkinsonism-dystonia, neurotransmitter, gene therapy, *SLC6A3*, DAT, iPSC

## Abstract

Infantile parkinsonism-dystonia due to dopamine transporter deficiency syndrome (DTDS) is an ultrarare childhood movement disorder caused by biallelic loss-of-function mutations in the *SLC6A3* gene. Advances in genomic analysis have revealed an evolving spectrum of *SLC6A3*-related neurological and neuropsychiatric disorders. Since the initial clinical and genetic characterisation of DTDS in 2009, there have been thirty-one published cases with a variety of protein-truncating variants (nonsense variants, splice-site changes, and deletions) and missense changes. Amino acid substitutions result in mutant proteins with impaired dopamine transporter function due to reduced transporter activity, impaired dopamine binding, reduced cell-surface expression, and aberrant posttranslational protein modification with impaired glycosylation. In this review, we provide an overview of the expanding clinical phenotype of DTDS and the precision therapies in development, including pharmacochaperones and gene therapy.

## 1. Introduction

The clinical spectrum of dopamine-related conditions is broad, encompassing movement disorders and neuropsychiatric diseases such as attention deficit hyperactivity disorder (ADHD), autism spectrum disorder (ASD), addiction, and bipolar disorder [1]. The dopaminergic system is involved in controlling the initiation of motion, reinforcement, and motivation, as well as contributing to emotion and cognitive functions (learning, attention, and memory) [1].

Within clinical neurology, symptoms indicative of dopamine dysregulation include bradykinesia, tremor, hypomimia and dystonia, neuropsychiatric features (apathy, anxiety, and impulse control disorders), and cognitive impairment, as observed in Parkinson’s disease [2]. Parkinsonism may also present in infancy, often in combination with dystonia; in contrast to adult patients, many affected children are found to have an underlying genetic aetiology, leading either to dopaminergic neurodegeneration or the impairment of dopamine synthesis, breakdown, or transport [3]. Several primary dopamine synthesis disorders are also reported, including tyrosine hydroxylase deficiency, aromatic L-amino acid dopa-decarboxylase (AADC) deficiency, pterin defects (GTPCH, SR, and PTPS), vesicular monoamine transporter (VMAT2) deficiency, and *DNAJC12* deficiency. These primary neurotransmitter disorders are associated with low cerebrospinal fluid dopamine metabolites [4]. Childhood disorders of dopaminergic deficiency may result from striatonigral neurodegeneration, as is evident in *DNAJC6*-related disease—one of many juvenile-onset forms of genetic parkinsonism—as well as numerous metabolic disorders, including the mitochondriocytopathies [5,6].

Dopamine transporter deficiency syndrome (DTDS) is a primary neurotransmitter disorder due to defective dopamine reuptake. Prior to gene discovery, the initial clinical description from 2004 reported three children presenting with infantile parkinsonism-dystonia and, paradoxically, raised cerebrospinal fluid (CSF) homovanillic acid (HVA) [7]. In 2009, with the identification of new patients—two cousins from consanguineous kindred and a separate singleton from another consanguineous family—further genetic analysis was possible. Through autozygosity mapping, the genetic interrogation of regions of homozygosity identified homozygous missense variants in *SLC6A3*, which encodes the dopamine transporter (DAT), in these two kindreds [8,9]. Since 2009, fifty-one patients have been identified worldwide with DTDS, with confirmed disease-causing biallelic variants in *SLC6A3*, which encodes DAT (Kurian personal communication).

DAT regulates dopamine homeostasis by transporting extracellular DA into the intracellular space that controls the synaptic levels of DA in the mesolimbic and nigrostriatal pathways [2]. In vitro modelling of DTDS-related *SLC6A3* variants revealed impaired dopamine transporter function due to reduced transporter activity, with impaired dopamine binding, reduced cell-surface expression, and aberrant posttranslational modification with impaired glycosylation [8]. The effects of failed dopamine reuptake result in persistent synaptic dopamine and raised HVA, with subsequent intraneuronal dopamine reduction and downregulation of TH activity through aberrant feedback to presynaptic D2 receptors, as observed in the DAT knockout mouse model [10]. DAT is the principal regulator of synaptic DA transmission, and genetic variants in *SLC6A3* alter expression, membrane localisation, hDAT density, dopamine reuptake activity, and dopamine neurotransmission dynamics, contributing to a pathophysiological spectrum from infantile to adult-onset parkinsonism-dystonia, termed DTDS. In addition, *SLC6A3* variants are associated with neuropsychiatric diagnoses, including autism spectrum disorder (ASD), attention deficit hyperactivity disorder (ADHD), and bipolar disorder [11,12,13,14]. In this review, we focus on the DTDS clinical spectrum, genetic variant modelling, and progress to precision therapeutics.

## 2. Clinical Spectrum of DTDS

DTDS is a primary neurotransmitter disorder that presents with infantile parkinsonism-dystonia due to biallelic loss-of-function mutations in the *SLC6A3* gene (OMIM # 613135). The detailed clinical characterisation of eleven children with DTDS established the classical features and progression of DTDS over time. Since 2011, there have been thirty-one DTDS patients reported in the literature [8,9,15,16,17,18,19,20,21,22,23,24], and a further unpublished twenty patients reported to our centre (Kurian personal communication).

### 2.1. Classical Early Onset DTDS

The classical DTDS phenotype typically manifests during early infancy with irritability, feeding difficulties, hypotonia, and delayed motor development [8,9,15,25]. A hyperkinetic movement disorder develops with features of chorea, dystonia, ballismus, and orolingual dyskinesia [8,9,15,25]. Over time, the condition evolves to parkinsonism-dystonia with the development of dystonic posturing, bradykinesia, distal tremor, rigidity, and hypomimia. The movement disorder progresses to akinesia in late childhood/early adolescence [8,9,15,25]. Some children also experience episodic status dystonicus and eye movement disorders with oculogyric crisis, ocular flutter, eyelid myoclonus, and saccade initiation failure [8,9,15,25]. Although the children present with a severe movement disorder with motor and speech delays, many individuals are reported to have relative preservation of intellect and good cognitive development [9,25]. Cognition in children with severe motor impairment, as seen in DTDS, is difficult to assess, but children with DTDS are thought to remain relatively cognitively intact, showing good working memory, communication with eye-gaze devices, accurate recognition of familiar information, and empathy and social awareness [9]. Secondary orthopaedic complications such as scoliosis and joint contractures are also described. Reported classical early onset DTDS individuals have a distinct CSF neurotransmitter analysis profile, with raised HVA (a stable metabolite of dopamine) and normal hydroxyindoleacetic acid levels (5-HIAA, a stable metabolite of serotonin). The HVA:HIAA ratio is raised at 5.0–13.0 (normal range: 1.0–4.0) [8,9,15]. MRI brain imaging can be normal or show subtle nonspecific abnormalities, such as mild delayed myelination, white matter abnormalities (such as periventricular leukomalacia), and prominence of external frontotemporal spaces. Single-photon emission computerised tomography (SPECT) with 123Ioflupane (DaTScan) imaging shows either absent or significantly reduced tracer uptake in the basal nuclei [9].

With over a decade of experience in treating early onset DTDS patients, it is evident that there is limited response to standard pharmacotherapies [9,15]. Palliative treatment endeavours to control symptoms; this includes the use of tetrabenazine and benzodiazepines to treat chorea and dyskinesia early in the disease, and dopamine agonists such as pramipexole and ropinirole [9,15,25]. There is limited or no response to levodopa treatment. Gabapentin has been useful in easing stiffness and reducing hyperkinesia in some cases (Kurian personal communication). Focal treatment with botulinum toxin injections for dystonia and orthopaedic intervention for severe contractures have been tried in some patients [9,15,25]. Episodes of status dystonicus can be life-threatening, with associated rhabdomyolysis requiring emergency intensive care management. Neurosurgical interventions such as deep brain stimulation and intrathecal baclofen have been used in older patients, with limited therapeutic benefit [9]. The associated bulbar dysfunction and feeding difficulties contribute to poor weight gain, and most patients require gastrostomy feeding. Affected patients are at significant risk of respiratory problems, such as chest infections and aspiration pneumonia; eight children have died from cardiorespiratory complications. Although the long-term outcome of DTDS remains to be fully characterised, it is clearly associated with a significant risk of morbidity and premature mortality, with a mean age of death of 10.4 years old (range: 3–16.2) [9,15] Kurian personal communication). The oldest surviving early onset DTDS patients are now in their fourth decade of life [11,21].

### 2.2. Atypical Later-Onset DTDS

Since the first description of early onset classical DTDS, there has been an expansion of the clinical phenotype, with the identification of patients with a milder, more slowly progressive condition associated with biallelic missense variants in *SLC6A3*. Such patients with later-onset atypical DTDS usually have a history of normal early neurodevelopment in infancy and early childhood (achieving independent ambulation and spoken language), presenting later in the second decade of life with tremor, progressive bradykinesia, dystonic posturing, and variable tone [15]. The first reported atypical DTDS kindred identified three brothers who presented at 10–11 years old with head tremor. The brothers were diagnosed at 16, 26, and 28 years old, respectively, with the older brothers developing progressive symptoms in their 20s with tremor affecting the head, arms, cervical dystonia and hypomimia, and speech deterioration [15]. All three individuals harboured homozygous missense variant Ala314Val, that on in vitro analysis retained 8% dopamine uptake activity, relatively higher than missense variants identified in classical infantile onset DTDS [15]. Another adult with atypical later-onset DTDS was reported; he initially presented with childhood failure to thrive as well as learning and behavioural problems in school. He presented with insidious right-sided tremor at 28 years old, which progressed to severe coarse tremor in all limbs and the trunk, with rigidity and bradykinesia, and was diagnosed with early onset PD at 35 years of age [16]. Molecular genetic investigation identified compound heterozygous missense variants Ile312Phe and Asp421Asn [16]. In vitro modelling of these variants showed both DAT variants exhibited markedly reduced dopamine uptake capacity (33% of the wildtype transporter) but preserved membrane targeting, consistent with impaired catalytic activity [16]. For DAT-Asp421Asn, substrate efflux experiments revealed a constitutive, anomalous efflux of dopamine, and electrophysiological analyses identified a large cation leak that might further perturb dopaminergic neurotransmission [16]. The long-term outcome of these individuals with atypical later-onset DTDS parkinsonism-dystonia is currently unknown.

## 3. Dominant-Negative *SLC6A3* Variants in Neurological and Neuropsychiatric Disease

Genetic variants in the *SLC6A3* gene affect different functional parameters of the transporter, including gene expression, glycosylation required for membrane trafficking, substrate affinity, reuptake activity, and transport direction. These differing facets of aberrant transporter function influence DA neurotransmission and contribute to a spectrum of neuropsychiatric DAT-related disorders that include ASD, ADHD, and bipolar disorder (Table 1) [11,12,13,14]. The in vitro and in vivo modelling studies of these missense variants associated with ASD, ADHD, and bipolar disorder suggest effects on dopamine efflux, endocytosis, and recycling, whilst missense variants associated with infantile DTDS reveal defects in expression, glycosylation, and impaired dopamine uptake (Table 2) [8,9,15].

More recently, dominant-negative variants in two unrelated individuals heterozygous for K619N have been identified [26], whereby the genetic mutation results in a protein that interferes with the normal function of the wildtype protein. Both patients were diagnosed with autism spectrum disorder (ASD). Patient 1 developed progressive right-sided tremor at 39 years old and was diagnosed with early onset parkinsonism at 41 years old with progressive loss of uptake activity, as measured with DaTScan. The second patient was diagnosed with ASD, but details of any movement disorder were not described. In vitro modelling of hDAT-K619N showed reduced dopamine uptake capacity, decreased surface expression, and accelerated cellular turnover on live fluorescence imaging [26]. Drosophila modelling showed age-dependent changes in locomotor response, with loss of hyperactive climbing response in hDAT-K619N-expressing flies by 23 days, and by day 30, the hDAT-K619N flies were showing deficient climbing compared to hDAT-WT-expressing flies [26]. Further in vivo modelling, using adeno-associated viral (AAV) vector-mediated unilateral expression in TH Cre mouse nigrostriatal neurons, revealed dominant-negative effects of hDAT-K619N that caused a dopamine uptake reduction to 40%, with a 30% reduction of total DAT observed [26]. These findings were accompanied with behavioural effects, with impaired amphetamine-induced rotations compared to wildtype [26]. The combination of cellular studies, drosophila modelling, and viral expression of hDAT-K619N in mice demonstrated a dominant-negative effect of the hDAT-K619N mutant. The dominant-negative effect of hDAT-K619N is likely linked to transporter oligomerization with the accelerated degradation of hDAT-K619N through interaction with hDAT-WT, resulting in the concomitant accelerated degradation of hDAT-WT [26,27]. The identification of these individuals adds to reports of heterozygous carriers of coding variants in *SLC6A3* associated with neuropsychiatric diseases, including bipolar disorder, ADHD, and autism spectrum disorder [14]. Together, these findings support a role for the dopamine transporter in modulating both motor and neuropsychiatric brain networks.

## 4. DTDS Molecular Genetics and Disease Mechanisms

**Figure 1:**
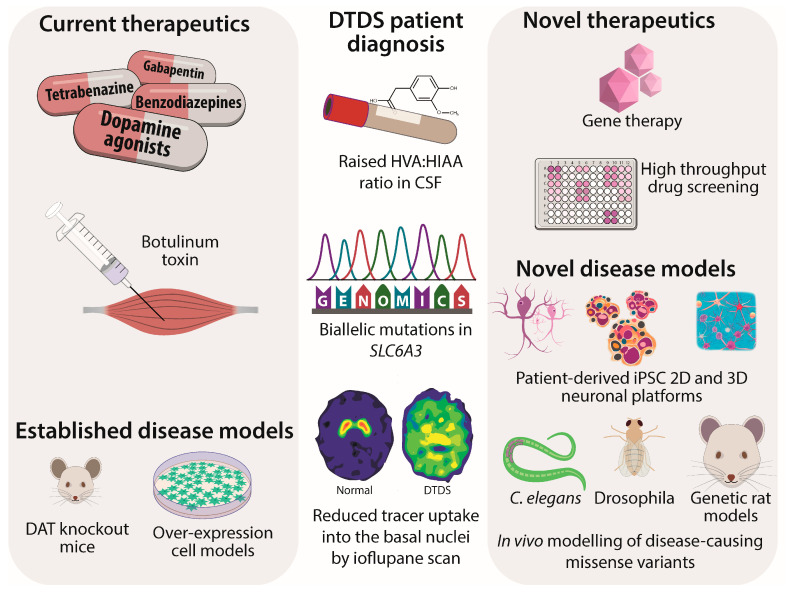
Schematic of DTDS current therapies, patient diagnosis, and novel therapeutics.

The genetic diagnosis of DTDS can be made through several genetic platforms, including whole-genome/exome analysis, gene panels, and targeted gene sequencing (Figure 1). A number of nonsense variants, splice-site changes, and frameshift deletions have been reported where nonsense-mediated decay or absent/truncated proteins are mechanistic factors in disease [8,9,15,16,17,18,19,20,21,22,23,24]. To date, twenty missense variants have been reported in DTDS (Table 2), with in vitro modelling undertaken for thirteen variants [8,9,15]. Mutant proteins have been shown to impair transporter function through (i) reduced transporter activity, (ii) impaired dopamine binding affinity, (iii) reduced cell-surface expression, and (iv) impaired glycosylation [8,9,15]. Where functional work was undertaken, all missense variants associated with early onset DTDS showed severely impaired residual dopamine uptake activity [8,9,15]. In vivo modelling of missense variants in *drosophila melanogaster* and *Caenorhabditis elegans* also corroborated DAT loss of function with abnormal motor activity [28,29].

## 5. Pharmacochaperone Therapy Development

Many mutations associated with classical DTDS have been shown to impair DAT cell-surface expression; mutant proteins appear to affect normal transporter folding and posttranslational modification, resulting in the retention of mutant DAT in the endoplasmic reticulum (ER), transporter mislocalisation with reduced cell-surface expression, and subsequent impairment of transport activity [30]. It is therefore not surprising that the role of pharmacochaperones in modifying DAT function has been evaluated. In one study of thirteen missense variants resulting in ER-retained DAT, in vitro modelling showed that the protein folding deficit exhibited by V158F, G327R, and L368Q was resolved by pharmacochaperones, noribogaine, or pifithrin-μ (heat shock protein 70), with the restoration of dopamine uptake [30]. These variants were also modelled in drosophila; whilst L368Q resulted in developmental lethality in drosophila, the V158F and G327R mutants showed reduced sleep duration, hyperlocomotion, and reduced head grooming [30]. Notably, there was a dose-dependent response to noribogaine and pifithrin-μ in V158F and G327R flies [30]. Ibogaine, the parent compound of noribogaine that binds to transporters (namely DATs), is potentially a pharmacochaperone that could be repurposed for DTDS, although its hallucinogenic side-effect profile may preclude its role in paediatric patients. Bhat et al. reconfigured the ibogaine ring system to generate several ibogaine analogues that were tested on thirteen DTDS missense variants. The best-performing novel pharmacochaperone was a tropane-based analogue that rescued 6 of the 13 missense variants (G386R, R521W, A314V, P395L, P554L, and R445C) [31]. Another study evaluated pharmacochaperones ibogaine and buproprion for L224P, R445C, and P529L in an in vitro model system [32]: pretreatment with ibogaine or buproprion restored cell-surface expression and dopamine uptake for A314V and R445C [32]. Pifithrin μ has also been evaluated in an induced pluripotent stem cell (iPSC) patient-derived midbrain dopaminergic neuronal model (with patient 1 homozygous for L368Q and patient 2 homozygous for P395L) [33]. Derived mature midbrain dopaminergic neurons at day 65 of differentiation were treated for 24 h with pifithrin-μ, before measuring the uptake of tritiated dopamine. Neurons derived from patient 1 (L368Q) showed a significant twofold increase in DAT activity, reaching 35% of the mean dopamine uptake activity observed in control lines, with no overall increase in total DAT protein [33]. No effect was observed in neuronal cultures derived from patient 2 (P395L). Overall, whilst there is significant therapeutic potential for pharmacochaperones in DTDS, studies to date suggest that the response is mutation-specific and not effective for all missense mutations. Furthermore, this approach would not be suitable for protein-truncating variants, which account for 16% DTDS variants identified to date.

## 6. Reducing DAT Lysosomal Degradation with Chloroquine

Previous studies have shown that chloroquine inhibits lysosomal activity and limits DAT lysosomal degradation [34]. For DTDS-associated variants, the ratio of mature glycosylated DAT to immature non-glycosylated DAT is shifted in vitro. As such, the immature form predominates, suggesting DAT degradation [8,9,15]. The effect of chloroquine has been evaluated in both cellular and drosophila models of the R445C mutant [34]. In vitro chloroquine treatment significantly increased the ratio of mature DAT to immature DAT in hDAT R445C-expressing cells and controls [35]. hDAT R445C drosophila showed significant motor deficits, with reduced movement vigour, reduced cumulative distance, time in fast movement, and increased flight initiation time. The supplementation of fly food with chloroquine for hDAT R445C drosophila significantly improved the time for flight initiation relative to vehicle [34], but had no significant effects in hDAT WT flies. These data suggest that chloroquine enhances DAT expression and improves flight initiation in hDAT R445C flies [34]. To date, chloroquine has not been evaluated for other DTDS variants, but remains an attractive treatment candidate, given that it could be repurposed and that it crosses the blood–brain barrier. However, given that long-term use is not routine clinical practice, further evaluation of the long-term safety and efficacy profile would be warranted before administration to DTDS patients.

## 7. Viral Gene Therapy Development

Viral gene therapies deliver therapeutic genes to affected cells, using modified viruses to transduce target cells and deliver a therapeutic gene. The main viral vectors utilised clinically for monogenic disease are adeno-associated virus and lentiviral vectors [35]. These transduce both mitotic and postmitotic cells and have been evaluated in clinical trials of adult Parkinson’s disease and the primary neurotransmitter disorder, AADC deficiency [36]. A gene therapy approach is ideally suited for early onset DTDS, as there is a well-defined midbrain target for gene supplementation with the wildtype *SLC6A3* gene to treat causative biallelic loss-of-function mutations. There have been two studies evaluating viral vector-mediated gene supplementation for DTDS. A proof-of-concept study delivered two AAV vectors into the midbrain of adult DAT knockout mice by stereotactic injection [37]. To achieve specificity for dopaminergic neurons, the first AAV expressed Cre recombinase under the control of the truncated rat TH promoter, and a second AAV contained murine DAT flanked by loxP sites, under the control of a constitutive CMV promoter. Cre recombinase expression thus permitted specific therapeutic DAT expression [37]. However, the potential neurotoxicity of Cre recombinase expression renders it clinically untranslatable. Our recent study evaluated gene therapy in a DTDS patient-derived iPSC neuronal model and a DAT knockout mouse model. We demonstrated that iPSC-derived midbrain dopaminergic (mDA) neurons exhibit neurodegeneration; this corroborates features of the DAT knockout mouse model and clinical progression observed in the patients [33]. Treating the patient neurons with lentiviral vector restored dopamine uptake and neuronal survival. We also undertook a proof-of-concept in vivo gene therapy study to treat neonatal mice. We injected AAV9 carrying human *SLC6A3* into the lateral ventricles of DAT knockout mice. This was well tolerated and rescued the survival, motor phenotype, and neurotransmitter profile. However, at high dosage, off-target DAT expression was associated with neurotoxic effects [33]. For clinical translation, we performed a midbrain stereotactic injection of AAV2 to restrict gene expression; this mode of injection and vector serotype has clinical precedent and achieves restriction of gene expression [33]. Our study showed dose-responsive restoration of motor activity and survival to wildtype levels with no off-target effects.

## 8. Conclusions

Since the first clinical report of infantile parkinsonism-dystonia with paradoxically raised dopamine metabolites, there have been profound advances in understanding DTDS. Advances in genomic analysis have transformed the diagnostic process for these children; the identification of the causative gene has facilitated the recognition of a distinct clinical syndrome, whilst increasing diagnostic access to next-generation sequencing technologies has prompted earlier diagnoses [12]. Such genomic advances have also allowed the recognition of broader clinical phenotypes associated with *SLC6A3*, encompassing DTDS parkinsonism-dystonia, early onset parkinsonism, and neuropsychiatric diseases (ADHD and ASD). Over the last decade, we have now identified 51 patients with DTDS. The ability to model disease-causing missense mutations in vitro has provided significant mechanistic insight. Our experience of evaluating DTDS patients confirms that the currently available treatment options are inadequate and palliative at best. Significant efforts to develop disease-modifying novel therapeutics for DTDS patients are now needed, and it is reassuring to know that approaches exploring the potential utility of novel pharmacochaperones and translational viral gene therapy approaches are underway. With the advent of new disease models such as 3D organoid modelling, it will be possible to better represent the brain microenvironment with various neuronal cell types and mimic the complexity of brain homeostasis in disease states to evaluate the effects of DAT dysfunction in human neuronal networks (Figure 1). Such humanised model systems will also provide a novel therapeutic platform for testing new experimental treatments. The ability to model DAT in multiple models in vitro and in vivo will prove invaluable to understanding disease mechanisms, the overlap between movement and neuropsychiatric phenotypes, and the therapeutic window—all with the ultimate aim of developing better precision medicines for DTDS patients.

## Figures and Tables

**Table 1:** SLC6A3 variants associated with neuropsychiatric disorders with predicted effects on proteins with in vitro modelling using cell overexpression systems.

Cases	Neuropsychiatric Diagnosis	*SLC6A3* Variant	Protein Effect	Functional Alteration hDAT	Vmax
1 [14]	ASD	Heterozygous c.1205C > T	Thr356Met	Outward bias: AMPH-DA efflux ↓efflux but constitutive effluxer	42 34
1 [14]	ADHD	Heterozygous c.1981C > T	Arg615Cys	Accelerated endocytosis and recycling Altered hDAT microdomain localisation	62 102
2 unrelated 21 patient cohort [14]	ASD + atypical DTDS, bipolar, depression schizophrenia	Heterozygous c.1995C > T	Lys619Asn	AMPH-DA efflux ↓ Mature ↓, surface ↓	69
5 unrelated [14]	Bipolar, ADHD, ASD	Heterozygous c.1814C > T	Arg559Val	AMPH-DA efflux ↓ Constitutive effluxer	100 104
2 siblings [14]	ASD	c.289C > T	Arg51W	AMPH-DA efflux ↓ Syntaxin interaction ↓	94

Autism spectrum disorder (ASD), Attention Deficit Hyperactivity Disorder (ADHD), Amphetamine-dopamine (AMPH-DA). Reduced = ↓.

**Table 2:** Summary of reported DTDS *SLC6A3* mutations with predicted effects on proteins with in vitro modelling using cell overexpression systems in the majority. Three variants were evaluated with in silico prediction effects only.

Case	Clinical	*SLC6A3* Variant	Protein Effect	Functional Effects on hDAT (In Vitro Modelling)	DAT Uptake %WT
1,2 [8,9]	Classical Homozygous	c.1103T→A	Leu368Gln	Mature ↓↓, surface ↓	0
3 [8,9]	Classical Homozygous	c.1184C→T	Pro395Leu	Mature ↓↓, surface ↓	0
4 [8,9]	Classical Homozygous	c.1156 + 5delG	Unknown— presumed splicing defect	N.D.	N.D.
5 [9]	Classical Compound heterozygous	c.472G→T	Val158Phe	Mature ↓↓, surface ↓	0
		c.1161C→T	Pro554Leu	Mature ↓↓, surface ↓	0
6 [9]	Classical Homozygous	c.1031+1G→A	Unknown— presumed splicing defect	N.D.	N.D.
7 [9]	Classical Homozygous	c.399delG	Ile134SerfX5	N.D.	N.D.
8 [9]	Classical Homozygous	c.1499_179del	Gly500GlufsX13	N.D.	N.D.
9 [9]	Classical Compound Heterozygous (3 variants)	c.979G→A	Gly327Arg	Surface ↓	0
		c.1315C→T	Gln439X	N.D.	N.D.
		c.1586C→T	Pro529Leu	Mature ↓↓, surface ↓	6.2
			Gly327Arg + Gln439X + Pro529Leu	N.D.	1.7
10 [9]	Classical Homozygous	c.671T→C	Leu224Pro	Mature ↓↓, surface ↓	0
11 [9]	Classical Homozygous	c.1561→T	Arg521Trp	Mature ↓, surface ↓ Syntaxin interaction ↓	26.9
12–14 [15]	Atypical Homozygous	c.941C→T	Ala314Val	Mature ↓, surface ↓	8.8
15 [15]	Classical Homozygous	c.1269 + 1G→A	Unknown— presumed splicing defect	N.D.	N.D.
16 [15]	Classical Homozygous	c.1269 + 1G→A	Unknown— presumed splicing defect	N.D.	N.D.
17 [15]	Classical Compound heterozygous	c.287-5_287-2delinsAAC	Unknown— presumed splicing defect	N.D.	N.D.
		c.1156G→A	Gly386Arg	Mature ↓↓	0
18 [15]	Classical Homozygous	c.1408_1409delins AG	Try470Ser	Mature ↓↓, surface ↓	0
19 [15]	Classical Compound heterozygous	c.254G→T	Arg85Leu	Mature ↓, surface ↓	0.5
		c.1333C→T	Arg445Cys	↓ Surface and block of N terminus release	5.6
			Arg85Leu + Arg445Cys		1.8
20 [16]	Atypical Compound heterozygous	c.934 A→T	Ile312Phe	Outward bias	56 (Vmax)
		c.1261 G→A	Asp421Asn	Inward bias: loss of Na^+^ Cl^−^ binding; constitute efflux	10
			Ile312Phe + Asp421Asn		30
21 [17]	Classical Homozygous	c.418_3800_653+3058del	Unknown— presumed splicing defect	N.D.	N.D.
22 [17]	Classical Homozygous	c.1156G→A	Gly386Arg	Mature ↓↓	0
23 [18]	Classical Homozygous	1139–1150del	Unknown— presumed splicing defect	N.D.	N.D.
24 [19]	Classical Homozygous	c.655G→A	Arg219Gly	In silico predicted damaged function	0 predicted
25 [20]	Classical Homozygous	c.655C→A	Arg219Ser	In silico predicted damaged function	0 predicted
26 [21]	Classical Homozygous	c.1639dupC	His547ProfsX56	N.D.	N.D.
27–29 [22]	Classical Homozygous	c.1333C→T	Arg445Cys	↓Surface and block of N terminus release	5.6
30 [23]	Classical Homozygous	c.1029→G	Tyr343X	N.D.	N.D.
31 [24]	Classical Homozygous	c.1297G→A	Gly433Arg	In silico predicted damaged function	0 predicted

N.D. = Not determined, as there is no hDAT product for genetic variant), reduced = ↓, severely reduced = ↓↓.

## Data Availability

No new data were created or analyzed in this study. Data sharing is not applicable to this article.
